# GSDME knockout alleviates hematopoietic stem cell irradiation injury and aggravates myeloid-biased differentiation

**DOI:** 10.3389/fcell.2025.1544320

**Published:** 2025-04-04

**Authors:** Lulu Su, Yinping Dong, Bowen Guan, Yuquan Wang, Yanhua Lu, Xinyue Wang, Wenxuan Li, Qidong Huo, Aimin Meng, Deguan Li

**Affiliations:** ^1^ Department of Oncology, Henan Provincial People’s Hospital, Zhengzhou University People’s Hospital, Henan University People’s Hospital, Zhengzhou, China; ^2^ Key Laboratory of Human Disease Comparative Medicine, Chinese Ministry of Health, Beijing Engineering Research Center for Laborator Animal Models of Human Critical Diseases, National Human Diseases Animal Model Resource Center, Institute of Laboratory Animal Sciences, Chinese Academy of Medical Sciences (CAMS) and Peking Union Medical College (PUMC), Beijing, China; ^3^ Tianjin Key Laboratory of Radiation Medicine and Molecular Nuclear Medicine, Institute of Radiation Medicine, Chinese Academy of Medical Sciences (CAMS) and Peking Union Medical College (PUMC), Tianjin, China

**Keywords:** GSDME, ionizing radiation, myeloid-biased differentiation, acute bone marrow suppression, long-term bone marrow injury

## Abstract

**Background:**

Bone marrow (BM) suppression is the most prevalent dose-limiting side effect of chemotherapy and radiotherapy. Exposure to ionizing radiation (IR) results in acute BM suppression and long-term BM injury. Gasdermin E (GSDME) is crucial for mediating apoptosis and pyroptosis during chemotherapy. However, its role in radiation-induced hematopoietic injury is not well established. Therefore, we aimed to investigate the role of GSDME on radiation-induced hematopoietic injury.

**Methods:**

We established hematopoietic radiation injury models in C57BL/6 mice and Gsdme^−/−^ mice. The peripheral blood (PB) counts, phenotypes of BM cells and spleen cells were analyzed. The colony-forming unit-granulocyte and macrophage assays and competitive repopulation assays were measured to evaluate the function of hematopoietic cells.

**Results:**

We demonstrated that GSDME regulates the survival and differentiation of hematopoietic stem cells (HSCs). The knockout of GSDME reduced the number and proliferation of HSCs and shortened the survival time of mice post IR. Additionally, GSDME knockout protected LSK (Lin-Sca1+c-kit+) cells, long-term HSCs (LT-HSCs), granulocyte–monocyte progenitors (GMPs), and myeloid cells (M cells) from IR injuries during acute BM suppression. Furthermore, GSDME knockout protected LSK cells, LT-HSCs, GMPs and M cells, alleviated the proliferation inhibition of hematopoietic progenitor cells (HPCs) and exacerbated lymphocyte damage during long-term BM injury.

**Conclusion:**

GSDME is vital for the survival and differentiation of HSCs, and its absence promotes myeloid-biased differentiation postirradiation. These findings highlight the critical role of GSDME in radiation-induced hematopoietic injury, particularly in the myeloid differentiation of HSCs.

## Introduction

Chemotherapy and radiotherapy for cancer frequently lead to bone marrow (BM) suppression, which is a major limiting side effect. Exposure to ionizing radiation (IR) not only causes acute BM suppression but also results in long-term BM injury (Mauch et al., 1995). Acute BM suppression occurs shortly after IR exposure and is primarily caused by the induction of hematopoietic cell apoptosis (Mohrin et al., 2010). In contrast, long-term BM injury is a latent condition that mainly involves hematopoietic stem cell (HSC) senescence, including impaired self-renewal, reduced long-term repopulating capacity, and myeloid skewing (Gardner et al., 2001). Long-term BM injury has limited potential for recovery and can lead to conditions such as hypoplastic anemia or myelodysplastic syndrome (van Os et al., 1998; Gardner et al., 2001; Wang et al., 2006). The prognosis and underlying mechanism of long-term BM injury are extremely poor.

Gasdermin E (GSDME) is a gene associated with autosomal dominant nonsyndromic hearing impairment and belongs to the gasdermin superfamily. GSDME is recognized as a promoter of pyroptosis and is activated via caspase-3-mediated cleavage to generate its N-terminal fragment. GSDME then induces secondary necrosis/pyroptosis by forming pores in the plasma membrane (Wang et al., 2017). In cells lacking GSDME, apoptosis occurs upon stimulation without progressing to necrosis, whereas those with high or moderate levels of GSDME directly undergo pyroptosis (Rogers et al., 2017).

The expression of GSDME varies across different cell types and tissues. Recent studies have demonstrated that GSDME plays a crucial role in the mechanism of tumor cell death induced by chemotherapy. This discovery is significant because it contributes to treatment efficacy and highlights the potential of GSDME as a valuable tool in chemotherapy. Several cancer cell lines that exhibit high levels of GSDME demonstrate activation of GSDME via caspase-3 following chemotherapy, resulting in a necrotic morphology characterized by plasma membrane swelling and lysis (Lu et al., 2018; Wang et al., 2018; Yu et al., 2019; Zhang et al., 2019). Notably, studies have shown that GSDME knockout impairs the therapeutic efficacy of ceritinib in NCI-H3122 cells, whereas GSDME knockdown attenuates the antitumor effect of cisplatin in oral squamous cell carcinoma (Lu et al., 2018; Wang et al., 2022). Similarly, GSDME expression is upregulated by decitabine, and its combination with chemotherapy or phototherapy enhances antitumor treatment efficacy. GSDME knockout has also been demonstrated to protect mice from chemotherapy-induced tissue damage and weight loss (Wang et al., 2017). Intriguingly, research suggests that GSDME is upregulated in response to glucocorticoid stimulation in T lymphoblastic leukemia CEM-C7 cells, resulting in the induction of pyroptosis and increased activation of caspase-3. Therefore, the expression of GSDME may play a vital role in the response of lymphoid malignancies to glucocorticoid treatment (Rogers et al., 2019).

Although a few studies have explored the association between GSDME and radiosensitivity, evidence suggests that GSDME-mediated pyroptosis occurs in colorectal cancer cells following radiation exposure. Additionally, GSDME-knockout mice exhibit protection against radiation-induced weight loss and tissue damage, indicating the influence of GSDME on colorectal cancer radiosensitivity and radiation-related toxicity to surrounding normal tissues through pyroptosis (Tan et al., 2022). Similarly, GSDME triggers irradiation-induced pyroptosis in nasopharyngeal carcinoma cells, and significantly reduced GSDME expression is observed in radioresistant nasopharyngeal carcinoma specimens. These findings establish low GSDME expression as a predictive factor for poorer prognosis and increased radioresistance in nasopharyngeal carcinoma (Di et al., 2022). As such, GSDME may prove to be a valuable asset in radiotherapy treatment. However, limited research has focused on the effects of GSDME on radiation-induced hematopoietic injury, and further investigation is warranted in this area.

The experiments described in this study aimed to observe hematopoietic changes in steady condition after GSDME knockout and then explore the effects of GSDME knockout in the BM and spleen during acute BM suppression and long-term BM injury. The results showed that GSDME knockout led to a decrease in the number and proliferation of HSCs and a decrease in the survival time of mice post-IR. GSDME knockout protected LSK (Lin^−^Sca1^+^c-Kit^+^) cells, long-term hematopoietic stem cells (LT-HSCs), granulocyte–monocyte progenitors (GMPs), and myeloid cells (M cells) and mitigated the inhibition of hematopoietic progenitor cells (HPCs) proliferation induced by IR during acute BM suppression and long-term BM injury. Additionally, GSDME knockout aggravated lymphocyte (LY) damage induced by IR during long-term BM injury. In summary, these findings suggest that GSDME plays a vital role in the survival and differentiation of HSCs. The knockout of GSDME can alleviate IR-induced HSC injury and aggravate the myeloid differentiation skew of HSCs.

## Materials and methods

### Mice

Gsdme^−/−^ mice (C57BL/6-Ly5.2, or 45.2) were kindly provided by the Feng Shao laboratory of the National Institute of Biological Sciences (Beijing, China). Gsdme^−/−^ mice were generated by comicroinjection of in vitro-translated Cas9 mRNA and guide RNAs into C57BL/6 zygotes, as described previously (Wang et al., 2017). Male C57BL/6-Ly5.2 mice were purchased from the Institute of Laboratory Animal Sciences, Chinese Academy of Medical Sciences & Peking Union Medical College. Male C57BL/6-Ly5.1 (or 45.1) mice were purchased from the Peking University Health Science Center. All the mice used in this study were between 8 and 12 weeks of age and weighed 21–24 g. All the experimental procedures were approved by the Animal Care and Ethics Committee at the Institute of Radiation Medicine, Chinese Academy of Medical Sciences & Peking Union Medical College (No. 1402).

### Peripheral blood (PB) and bone marrow mononuclear cell (BM-MNC) counts

For the analysis of PB counts, PB from the orbital sinus was collected and analyzed on a hematology analyzer (MEK-7222K, Japan). Total bone marrow cells (BMCs) were flushed from mouse femurs with sterile phosphate buffer solution, and BM-MNCs were harvested as previously described (Li et al., 2013; Chang et al., 2015). The number of viable BMCs was counted using a hematology analyzer and expressed as ×10^7^/femur.

### BM-MNCs and mouse treatment

BM-MNCs were divided into three groups: (a) the control group, (b) the 2 Gy group, and (c) the 4 Gy group. BM-MNCs were irradiated with X-rays at a dose rate of 1.08 Gy/min using an RS2000 (Rad Source Technologies). Male C57BL/6-Ly5.2 mice and Gsdme^−/−^ mice were also divided into three groups: (a) the control group; (b) the 2 Gy group; and (c) the 4 Gy group. Mice from the 2 Gy and 4 Gy groups were exposed to 2 or 4 Gy γ-rays. Male C57BL/6-Ly5.2 mice and Gsdme^−/−^ mice were exposed to a lethal dose (7.5 Gy) of total body irradiation (TBI) for the survival study. Male C57BL/6-Ly5.1 mice were exposed to a lethal dose (two doses of 4 Gy, separated by a 4 h interval) of TBI for the competitive transplantation assay (CRA). Mice were irradiated using a 137Cs source housed in an Exposure Instrument Cammacell-40 (Atomic Energy of Canada Lim).

### Colony-forming unit-granulocyte and macrophage (CFU-GM) assays

*In vitro*, BM-MNCs were cultured under aseptic conditions in RPMI-1640 (HyClone) supplemented with 10% fetal bovine serum (Gibco) and penicillin (100 units/mL)/streptomycin (100 μg/mL) (Gibco) in 5% CO2 at 37°C prior to irradiation. BM-MNCs were plated into 24-well plates and irradiated with the required doses (0 Gy, 2 Gy, or 4 Gy) of X-rays for the CFU-GM assays. *In vivo*, both wild-type (WT) and Gsdme^−/−^ mice were exposed to the required doses (0 Gy, 2 Gy, and 4 Gy) of γ-ray irradiation for CFU-GM assays. CFU-GM assays were conducted by culturing BM-MNCs or BMCs in MethoCult GF M3534 methylcellulose medium (STEMCELL Technologies). The colonies of CFU-GM were counted on day 7 following the manufacturer’s protocol. Only colonies with more than 30 cells were included in the count.

### Histological and pathological analysis

For pathological analysis, spleens from control and irradiated mice were fixed with 4% formalin, embedded in paraffin, sectioned, and stained with hematoxylin and eosin.

### Cell isolation, staining and flow cytometry

Single-cell suspensions were prepared from BM or spleens of control and irradiated mice. The cells were stained with fluorochrome-conjugated antibodies purchased from eBioscience or BD Biosciences (listed in Supplementary Table 1). Flow cytometry analysis was performed using a Gallios flow cytometer (Beckman) and BD Accuri C6 instrument (BD Biosciences). The frequencies of LSK cells (Lin^−^Sca1^+^c-Kit^+^), HPCs (Lin^−^Sca1^−^c-Kit^+^), LT-HSCs (Lin^−^Sca1^+^c-Kit^+^CD34^−^), short-term HSCs (ST-HSCs) (Lin^−^Sca1^+^c-Kit^+^CD34^+^), common myeloid progenitors (CMPs) (CD34^+^CD16/32^−^Lin^-^Sca1^−^c-Kit^+^), M cells in BM (CD11b^+^Gr-1^+^), M cells in spleens (CD11b^+^), NE cells in spleens (CD11b^+^Gr-1^+^), T cells (CD3e^+^) and B cells (B220^+^), common lymphoid progenitors (CLPs) (CD127^+^Lin^−^Sca1^+^c-Kit^+^), GMPs (CD34^+^CD16/32^+^Lin^−^Sca1^−^c-Kit^+^) and megakaryocytic-erythrocytic progenitors (MEPs) (CD34^−^CD16/32^−^Lin^-^Sca1^−^c-Kit^+^) were analyzed.

### CRA

CRA was performed by transplanting a 5:1 ratio of 45.2 donor cells to 45.1 competitive cells into 4 Gy-irradiated recipients. After stable hematopoietic reconstitution (2 months), mice were sacrificed, and BMCs were harvested (Shao et al., 2014). For the first transplantation, BMCs (1 × 10^6^) were pooled from three CD45.2 mice after they were exposed to a sublethal dose (4 Gy) of TBI or sham irradiated either immediately or 4 weeks postirradiation as donor cells. The cells were mixed with 2 × 10^5^ competitive BMCs pooled from three CD45.1 mice and then transplanted into lethally irradiated (8 Gy TBI) CD45.1 recipients (5–6 recipients/group) via lateral canthus vein injection. To analyze the engraftment of donor cells, peripheral blood was harvested at 4 and 8 weeks, and BMCs were harvested at 8 weeks after the first transplantation. Single-cell suspensions of PB and BM were prepared and the cells were stained with fluorochrome-conjugated antibodies. The self-renewal and differentiation capacities of donor-derived HSCs from bone marrow were then analyzed.

### Statistical analysis

SPSS19.0 software was utilized for conducting the statistical analyses. Unpaired Student’s t-test was used to compare two groups of normally distributed data with homogenous variance. For multiple groups, one-way analysis of variance with Tukey’s *post hoc* comparison was performed when the data followed a normal distribution. All the data are expressed as the mean ± standard error of the mean (SEM) of at least three independent experiments. Statistical significance was determined at *P* < 0.05. The survival curves for 30 days were compared using Kaplan-Meier methods and a log-rank test. The sample sizes for all the experiments were determined through calculations based on prior experience. Animals were randomly assigned during collection, and the strain, sex, and age of the mice were consistent. The data analysis was conducted in a blinded manner. However, during the experiment and outcome assessment, the investigators were aware of the group allocation. Graphs showing in the results were produced by PRISM 5.0.

## Results

### Knockout of HSCs impaired the HSC pool in the BM under steady conditions

**FIGURE 1:**
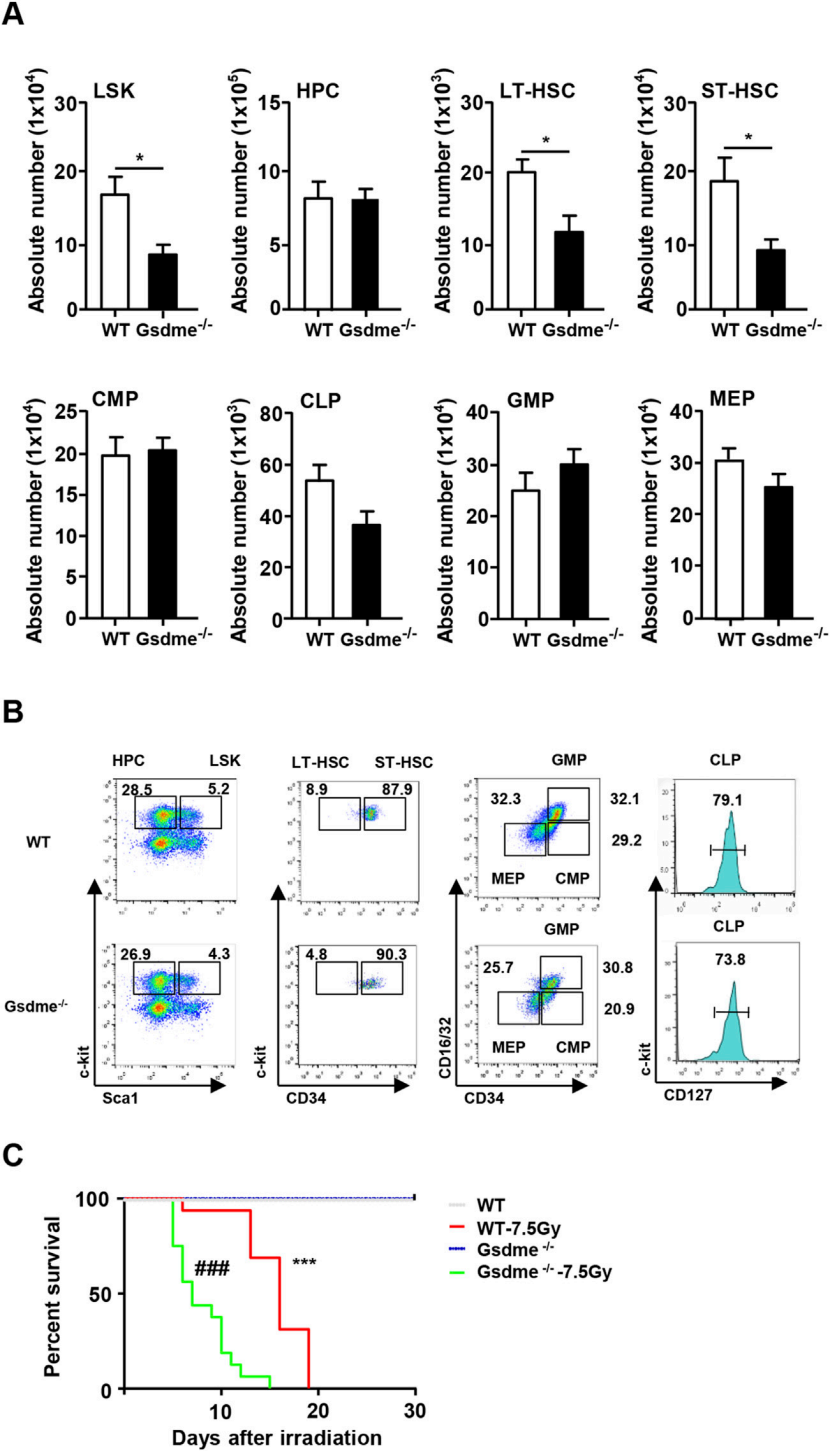
Knockout of GSDME impaired HSCs pool in BM in steady condition. WT and Gsdme^−/−^ mice were used at 8–12 weeks to obtain BMCs. Single-cell suspensions of BM were prepared and cells were stained with fluorochrome-conjugated antibodies. **(A)** The absolute numbers of LSK, HPC, LT-HSC, ST-HSC, CMP, CLP, GMP and MEP in BM of WT and Gsdme^−/−^ mice, n = 6, *P < 0.05. **(B)** Gating strategy for isolation of LSK, HPC, LT-HSC, ST-HSC, CMP, CLP, GMP and MEP in WT and Gsdme^−/−^ BM. **(C)** Kaplan-Meier survival curve of WT and Gsdme^−/−^ mice that underwent lethal irradiation (7.5Gy), n = 16, ***P < 0.001 WT mice post IR compared with WT mice, ^###^ *P* < 0.001 Gsdme^−/−^ mice post IR compared with Gsdme^−/−^ mice. Data are expressed as mean ± SEM.

To assess the significance of GSDME in the hematopoietic system, the cellular composition of BM hematopoietic cells was initially examined. There was no significant difference in the number of BMCs (*P* > 0.05) (Supplementary Figure 2A). Furthermore, the absolute numbers of LSKs (*P* = 0.015), LT-HSCs (*P* = 0.025) and ST-HSCs (*P* = 0.024) were significantly reduced in the BM of Gsdme^−/−^ mice (Figure 1A). Gating strategy for isolation of LSK, HPC, LT-HSC, ST-HSC, CMP, CLP, GMP and MEP in WT and Gsdme-/- BM was displayed (Figure 1B). However, there were no changes in the absolute numbers of other progenitor populations, including HPCs (*P* = 0.921), CMPs (*P* = 0.816), CLPs (*P* = 0.068), GMPs (*P* = 0.433) and MEPs (*P* = 0.147), in the BM of either Gsdme^−/−^ or WT mice (Figure 1A). Overall, these findings indicated that the knockout of GSDME resulted in an impaired HSC pool under steady conditions.

Subsequently, the mice were exposed to a lethal dose of TBI to evaluate the influence of GSDME on mouse survival. All mice that received 7.5 Gy irradiation died by day 15. Interestingly, Gsdme^−/−^ mice exhibited a shorter survival time, consistent with the decreased numbers of HSCs (Figure 1C). These results indicated that GSDME plays a significant role in the radiosensitivity of mice, and its knockout led to a decrease in the survival rate following TBI.

To further explore the differential effect of IR on cell viability and CFU-GM *in vitro*, we isolated BM-MNCs from Gsdme^−/−^ and WT mice and cultured them *in vitro* with various doses of radiation. Interestingly, IR (2, 4 Gy) inhibited the cell viability of BM-MNCs and the proliferation of HPCs *in vitro*. However, no differences were detected between Gsdme^−/−^ and WT mice (Supplementary Figure 1). These findings demonstrated that the knockout of GSDME didn’t influence the radiation-induced decrease in cell viability or HPC function *in vitro*.

In conclusion, GSDME knockout increased the radiosensitivity of mice by reducing the number of HSCs, resulting in a significant decrease in the survival time of mice after IR. These findings suggest that GSDME strongly affects the genomic stability of hematopoietic cells.

### GSDME knockout protected LSK, LT-HSC, GMP, M cells and the proliferation of HPC during the stage of acute BM suppression

**FIGURE 2:**
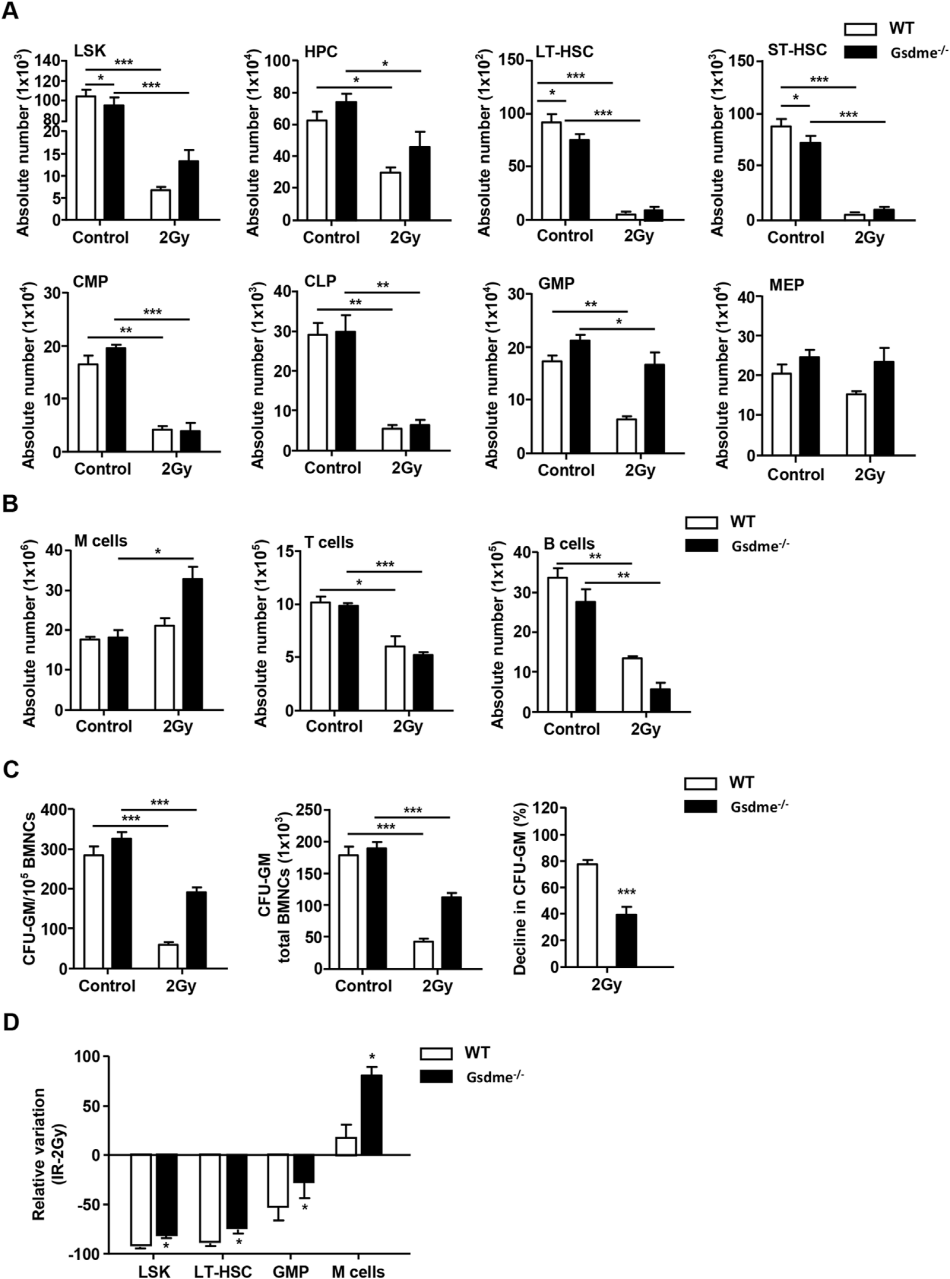
GSDME knockout protected LSK, LT-HSC, GMP, M cells and the proliferation of HPC during the stage of acute BM suppression. WT and Gsdme^−/−^ mice were exposed to IR at doses of 0 Gy (control) or 2 Gy. The BMCs were collected at 1 week after IR and in controls. Single-cell suspensions of BM were prepared and the cells were stained with fluorochrome-conjugated antibodies. **(A)** Absolute numbers of LSK, HPC, LT-HSC, ST-HSC, CMP, CLP, GMP and MEP in WT and Gsdme^−/−^ BM, n = 3. **(B)** Absolute numbers of M cells, T cells and B cells in WT and Gsdme^−/−^ BM, n = 3. **(C)** BMCs were cultured in MethoCult GF M3534 methylcellulose medium for 7 days, and then analyzed the CFU-GM. Data were from 1 experiment with 3 mice and 6 biologic replicates. **(D)** The relative variation of LSK, LT-HSC, GMP and M cells between irradiation and WT group, n = 3. Data are expressed as mean ± SEM, *P < 0.05; **P < 0.01; ***P < 0.001.

To gain deeper insight into the role of GSDME in hematopoiesis during the stage of acute BM suppression, the phenotypes of hematopoietic cells were detected. After mice were exposed to 2 Gy IR post 1 week, the BMCs were collected and analyzed. The results revealed GSDME knockout had no protection on absolute numbers of HPC, ST-HSC, CMP, CLP, MEP, T cells and B cells in BM after 2Gy TBI (Figures 2A, B). Interestingly, the LSK (*P* = 0.02), LT-HSC (*P* = 0.01) and GMP (*P* = 0.02) in Gsdme^−/−^ mice BM showed a lower decrease under the acute conditions post IR (Figure 2D). GSDME knockout could significantly increase the absolute number of M cells (*P* = 0.01) in BM (Figure 2D). Remarkably, the results showed the proliferation ability of HPC in Gsdme^−/−^ mice was stronger than that of WT mice HPC, which indicating that GSDME knockout could mitigate the proliferation inhibition of HPC after IR (Figure 2C). These results suggested GSDME knockout protected LSK, LT-HSC, GMP, M cells and the proliferation of HPC during the stage of acute BM suppression.

### Gsdme^−/−^ mice displayed extramedullary hematopoiesis in spleens during the stage of acute BM suppression

**FIGURE 3:**
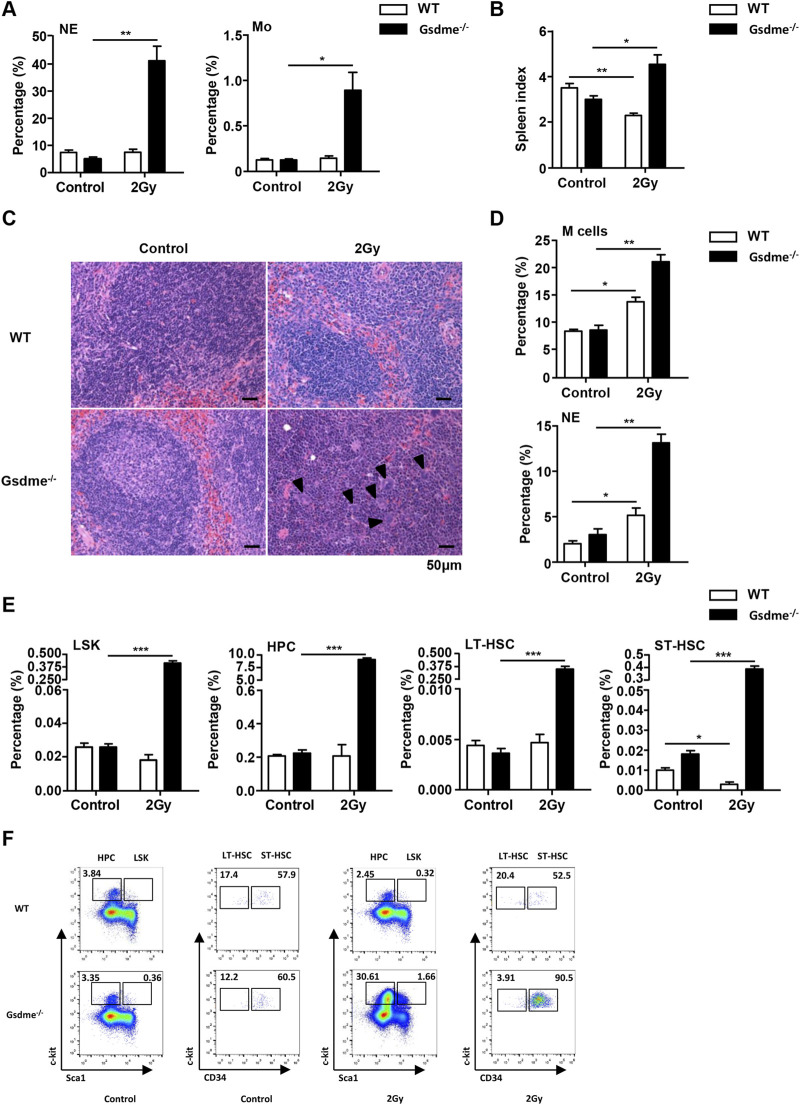
Gsdme^−/−^ mice displayed extramedullary hematopoiesis in spleens during the stage of acute BM suppression. WT and Gsdme^−/−^ mice were exposed to IR at doses of 0 Gy (control) or 2 Gy. The PB and spleens were collected at 1 week after IR and in controls. Single-cell suspensions of PB and spleens were prepared. Cells of PB and spleens were stained with fluorochrome-conjugated antibodies. **(A)** Percentages of NE and Mo in WT and Gsdme^−/−^ PB, n = 3. **(B)** The spleen index in WT and Gsdme^−/−^ mice, n = 3. **(C)** HE staining of the spleen from the WT and Gsdme^−/−^ mice (bar: 50 µm). Arrowheads indicated megakaryocytes. **(D)** Percentages of M cells and NE in the spleens from WT and Gsdme^−/−^, n = 3. **(E)** Percentages of LSK, HPC, LT-HSC and ST-HSC in WT and Gsdme^−/−^ spleens, n = 3. **(F)** Gating strategy for isolation of LSK, HPC, LT-HSC, and ST-HSC in WT and Gsdme^−/−^ spleens. Data are expressed as mean ± SEM, *P < 0.05; **P < 0.01; ***P < 0.001.

To assess the effect of GSDME on extramedullary hematopoiesis in spleens during the stage of acute BM suppression, the spleen index, histopathologic examination and phenotype were detected after mice were exposed to 2 Gy IR post 1 week. Physical examinations revealed that Gsdme^−/−^ mice displayed higher spleen indexes than WT mice (Figure 3B). Consistently, histopathologic examination showed evident extramedullary hematopoiesis, including clustered megakaryocytes (arrowheads) in Gsdme^−/−^ mice post 2 Gy IR (Figure 3C). Flow cytometry analyses confirmed splenic extramedullary hematopoiesis during the stage of acute BM suppression induced by 2 Gy IR in Gsdme^−/−^ mice, characterized by a remarkable increase in percentages of M cells, LSK, HPC, LT-HSC and ST-HSC in Spleen (Figures 3D–F), in agreement with NE and Mo expansion in PB compared to WT mice (Figure 3A). In addition, the percentages of LY in Gsdme^−/−^ mice PB showed a greater decrease under the acute conditions post IR (Supplementary Figure 2A). However, no statistically significant difference was observed in the WBC, RBC, HGB and PLT between WT and Gsdme^−/−^ mice during the stage of acute BM suppression (Supplementary Figure 2A). Collectively, these results demonstrated that Gsdme^−/−^ mice displayed extramedullary hematopoiesis in spleens to compensate the hematopoiesis defect during the stage of acute BM suppression.

### GSDME knockout had protective effects on LSK, LT-HSC, GMP, M cells and the proliferation of HPC during the stage of long-term BM injury

**FIGURE 4:**
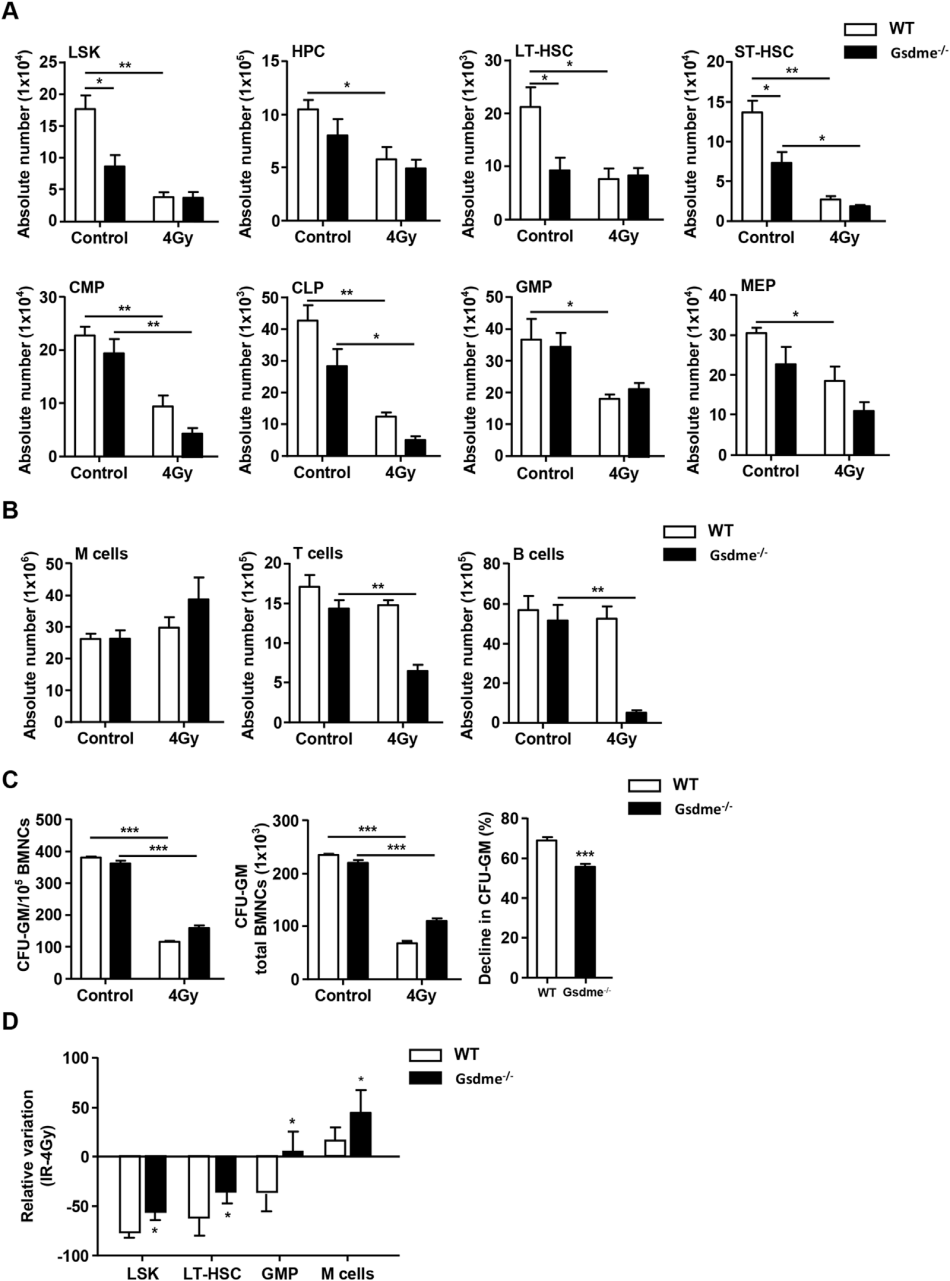
GSDME knockout had a protective effect on LSK, LT-HSC, GMP, M cells and the proliferation of HPC during the stage of long-term BM injury. WT and Gsdme^−/−^ mice were exposed to IR at doses of 0 Gy (control) or 4 Gy. The BMCs were collected at 4 weeks after IR and in controls. Single-cell suspensions of BM were prepared and the cells were stained with fluorochrome-conjugated antibodies. **(A)** Absolute numbers of LSK, HPC, LT-HSC, ST-HSC, CMP, CLP, GMP and MEP in WT and Gsdme^−/−^ BM, n = 3. **(B)** Absolute numbers of M cells, T cells and B cells in WT and Gsdme^−/−^ BM, n = 3. **(C)** BMCs were cultured in MethoCult GF M3534 methylcellulose medium for 7 days, and then analyzed the CFU-GM. Data were from 1 experiment with 3 mice and 6 biologic replicates. **(D)** The relative variation of LSK, LT-HSC, GMP and M cells between irradiation group and WT group, n = 3. Data are expressed as mean ± SEM, *P < 0.05; **P < 0.01; ***P < 0.001.

To explore the role of GSDME in hematopoiesis during the stage of long-term BM injury, the phenotype in hematopoietic cells were monitored after mice were exposed to 4 Gy IR post 4 weeks. The results revealed that LSK (*P* = 0.011), LT-HSC (*P* = 0.036), GMP (*P* = 0.040) and M cells (*P* = 0.019) of Gsdme^−/−^ mice showed a higher survival rate after exposure to 4-Gy radiation than WT mice (Figure 4D). Additionally, GSDME knockout had slight protection on the survival of HPC and MEP post IR (Figure 4A). However, GSDME knockout had no protection on the absolute numbers of ST-HSC, CMP, CLP, T cells and B cells in BM (Figures 4A, B). T cells and B cells in BM of Gsdme^−/−^ mice showed a lower survival after 4Gy radiation than WT mice, which indicating that GSDME knockout could aggravate the damage of LY after irradiation during the long-term BM injury (Figure 4B). Meanwhile, these results showed the proliferation ability of HPC in Gsdme^−/−^ mice was higher than that of WT HPC after 4-Gy radiation, which indicating GSDME knockout could mitigate the proliferation inhibition of HPC (Figure 4C). Collectively, these results demonstrated GSDME knockout could improve the radiation resistance of LSK, LT-HSC, GMP and M cells, while aggravating myeloid-biased differentiation and proliferation after irradiation during the stage of long-term BM injury. All these findings indicated that GSMDE plays a crucial role in the regulation of HSC differentiation.

### GSDME knockout slightly affects extramedullary hematopoiesis in spleens during the stage of long-term BM injury

**FIGURE 5:**
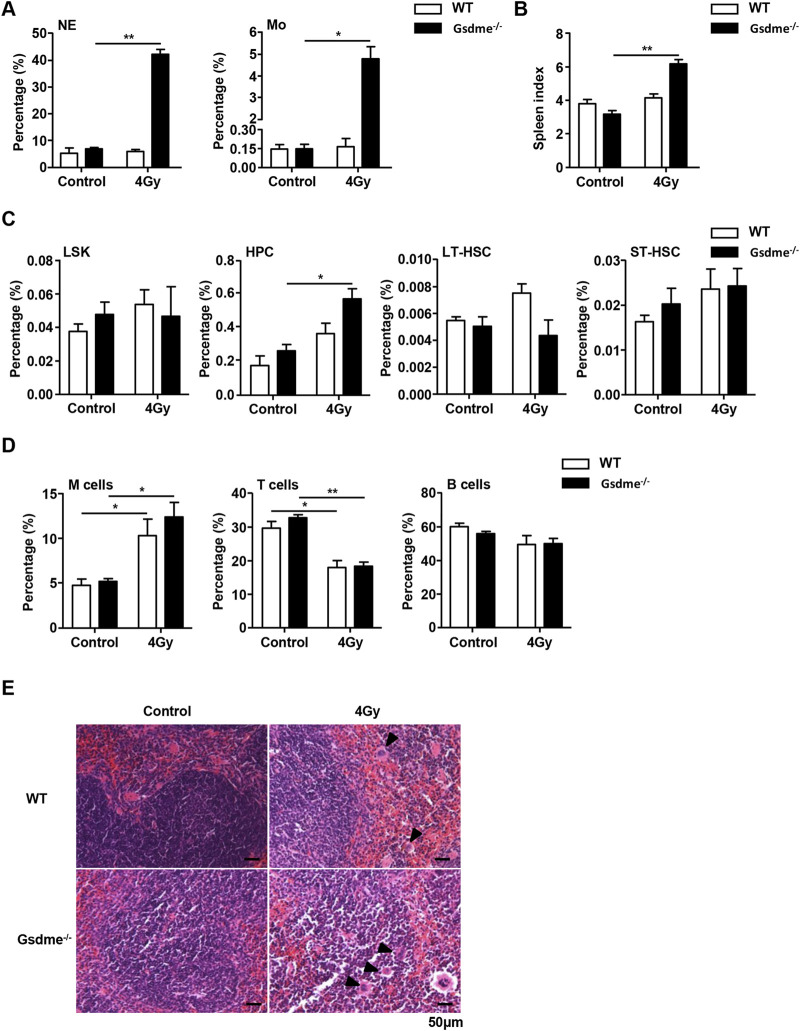
GSDME knockout slightly affects extramedullary hematopoiesis in spleens during the stage of long-term BM injury. WT and Gsdme^−/−^ mice were exposed to IR at doses of 0 Gy (control) or 4 Gy. The PB and spleens were collected at 4 weeks after IR and in controls. Single-cell suspensions of PB and spleens were prepared. Cells of PB and spleens were stained with fluorochrome-conjugated antibodies. **(A)** Percentages of NE and Mo in the PB from WT and Gsdme^−/−^ mice, n = 3. **(B)** The spleen index in WT and Gsdme^−/−^ mice, n = 3. **(C)** Percentages of LSK, HPC, LT-HSC and ST-HSC in WT and Gsdme^−/−^ spleens, n = 3. **(D)** Percentages of M cells, T cells and B cells in WT and Gsdme^−/−^ spleens, n = 3. **(E)** HE staining of the spleen from the WT and Gsdme^−/−^ mice (bar: 50 µm). Arrowheads indicated megakaryocytes. Data are expressed as mean ± SEM, *P < 0.05; **P < 0.01.

To address the impact of GSDME on extramedullary hematopoiesis in spleens during the stage of long-term BM injury, the spleen index, histopathologic examination and phenotype were investigated after mice were exposed to 4 Gy IR post 4 weeks. Physical examinations showed that Gsdme^−/−^ mice displayed higher spleen indexes than WT mice 4 weeks after irradiation (Figure 5B). Concomitantly, histopathologic examination revealed extramedullary hematopoiesis, including clustered megakaryocytes (arrowheads) in Gsdme^−/−^ mice after IR (Figure 5E). Furthermore, flow cytometry analyses revealed splenic extramedullary hematopoiesis during the stage of long-term BM suppression in Gsdme^−/−^ mice, characterized by an increased percentage of HPC (Figure 5C), in agreement with NE and Mo expansion in PB in comparison with WT mice (Figure 5A). In addition, the percentages of LY in Gsdme^−/−^ mice PB showed a greater decrease during the stage of long-term BM suppression (Supplementary Figure 2B). However, results showed GSDME knockout had no effect on the frequencies of LSK, LT-HSC, ST-HSC, M cells, T cells and B cells in spleens after 4 Gy IR (Figures 5D, E). Additionally, GSDME deficiency had no effect on the decrease of most PB cells (including WBC, RBC, HGB and PLT) and BMCs during the stage of long-term BM injury (Supplementary Figure 2B). Thus, these data indicated that GSDME knockout had only a slight effect on extramedullary hematopoiesis in spleens during the stage of long-term BM injury.

### GSDME knockout had no protective effect on the HSC long-term and multilineage engraftment reduction induced by IR

**FIGURE 6:**
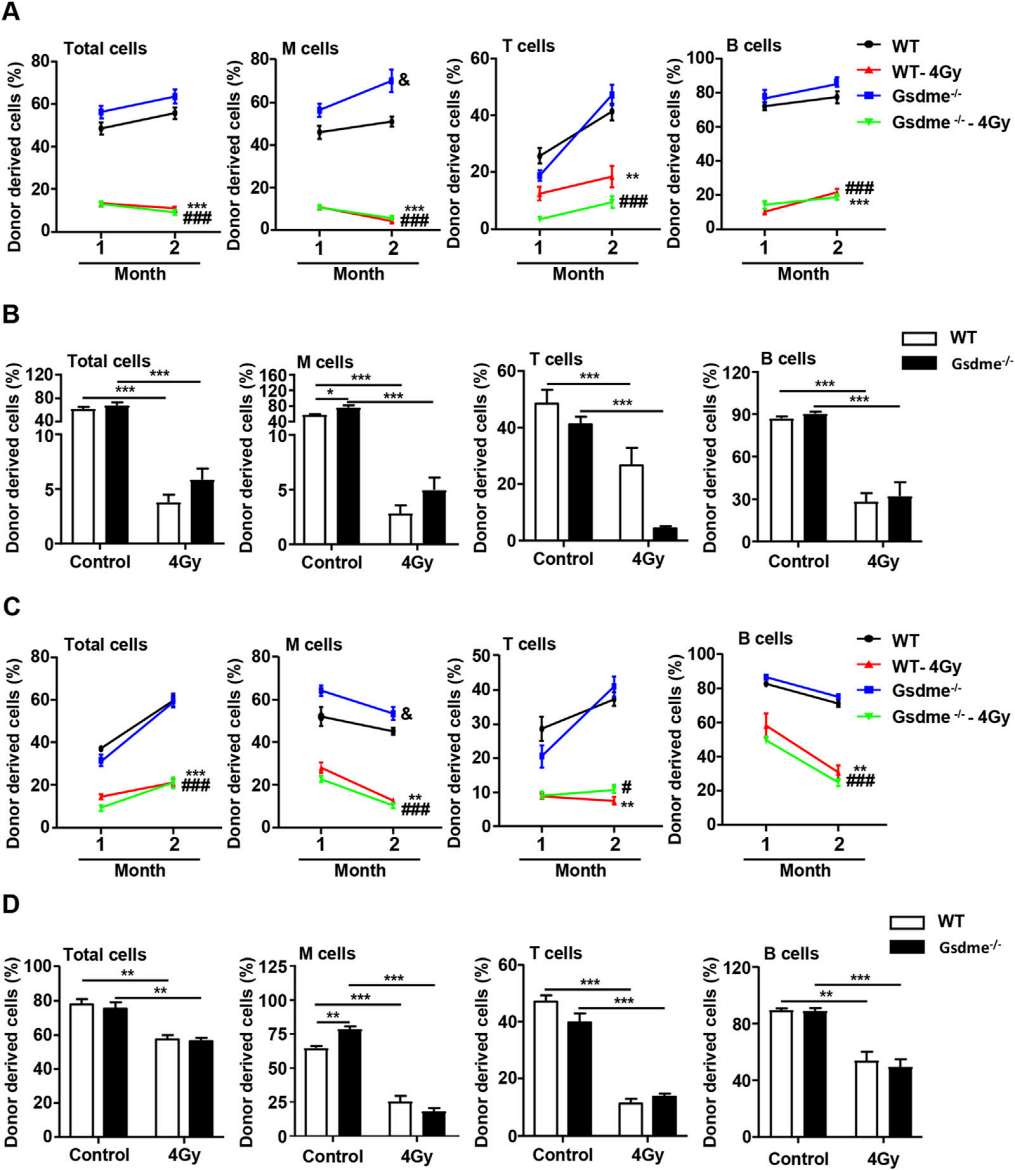
GSDME knockout had no protective effect on the HSCs long-term and multilineage engraftment reduction induced by IR. Competitive transplantation assays were performed by transplanting a 5:1 ratio of 45.2 donor cells to 45.1 competitive cells into 4 Gy-irradiated recipients. After stable hematopoietic reconstitution (2 months), mice were sacrificed, and BMCs were harvested. The PB chimera for total cells, M cells, T cells, and B cells were assessed after 1 and 2 months. **(A)** Donor chimerism of PB in primary recipients transplanted with 2 × 10^6^ BMCs from WT or Gsdme^−/−^ mice with or without 4 Gy IR immediately. Percentages of total cells, M cells, T cells, and B cells in PB of recipient mice at 1 and 2 months after transplantation, n = 5, ^&^ *P* < 0.05 Gsdme^−/−^ mice compared with the WT mice; **P < 0.01, ***P < 0.001 WT mice post IR compared with WT mice; ^###^ *P* < 0.001 Gsdme^−/−^ mice post IR compared with Gsdme^−/−^ mice. **(B)** Percentages of total cells, M cells, T cells, and B cells in BM of recipient mice, described in **(A)** at 2 months post-transplantation, n = 5, *P < 0.05; ***P < 0.001. **(C)** Donor chimerism of PB in primary recipients transplanted with 2 × 10^6^ BMCs from WT or Gsdme^−/−^ mice at 4 weeks with or without 4 Gy IR. Percentages of total cells, M cells, T cells, and B cells in PB of recipient mice at 1 and 2 months after transplantation, n = 6, ^&^ *P* < 0.05 Gsdme^−/−^ mice compared with the WT mice; **P < 0.01, ***P < 0.001 WT mice post IR compared with WT mice; ^#^ *P* < 0.05, ^###^ *P* < 0.001 Gsdme^−/−^ mice post IR compared with Gsdme^−/−^ mice. **(D)** Percentages of total cells, M cells, T cells, and B cells in BM of recipient mice, described in **(C)** at 2 months post-transplantation, n = 6, **P < 0.01; ***P < 0.001. Data are expressed as mean ± SEM.

To further dissect the differentiation ability of HSCs, CRA were performed. The chimerism in PB was analyzed 1 and 2 months after transplantation. The recipient mice were sacrificed at 2 months after transplantation and the chimerism in BM was also analyzed. The donor cells accepted sham irradiation remained stable engraftment in Gsdme^−/−^ and WT mice (Figures 6A-D). The percentage of M cells in Gsdme^−/−^ BM was higher than that in WT BM at 1 and 2 months after transplantation, suggesting that GSDME knockout could improve the differentiation and proliferation of M cells (Figures 6B, D). Additionally, all lineages derived from Gsdme^−/−^ and WT cells after expose to 4 Gy IR were both significantly reduced in both PB and BM after transplantation (Figures 6A-D). However, the percentage of T cells in Gsdme^−/−^ BM after expose to 4 Gy IR immediately was significantly lower than that in WT BM at 2 months after transplantation (Figure 6A), indicating that GSDME knockout could aggravate the damage of LY post IR in the early stage of radiation. These findings indicated that exposure to a sublethal dose of TBI immediately or 4 weeks post IR (4 Gy) could cause long-term BM injury. However, GSDME knockout had no protective effect on the HSC long-term and multilineage engraftment reduction induced by IR.

## Discussion

The toxic effect of radiotherapy on normal tissues is the major limitation for its clinic application for cancer treatment. Long-term cancer survivors are at risk for developing IR-induced long-term BM injury. Recent studies have identified GSDME as a vital factor in mediating the toxicity of chemotherapeutic agents to normal tissues by inducing pyroptosis (Wang et al., 2017; Yu et al., 2019). The expression of GSDME also determined radiosensitivity in colorectal cancer and nasopharyngeal carcinoma (Di et al., 2022; Tan et al., 2022). However, the role of GSDME in radiation-induced hematopoietic injury has not been investigated. Gene chip results showed that GSDME expression was downregulated in LSK 1 month after 6 Gy TBI (data not shown). In this study, using Gsdme^−/−^ mice, for the first time we found GSDME was important in genomic stability of hematopoietic cells. GSDME knockout exerted a protective effect on radiation-induced hematopoietic injury, especially for LSK, LT-HSC, GMP, and M cells, and the proliferation of HPC. GSDME knockout may also be related to myeloid differentiation skew. These results suggested GSDME might be a critical determining factor of radiosensitivity and serve as a potential target for radiation protection.

To date, there is limited information on the role of GSDME in the radiosensitivity of hematopoietic cells. Nonetheless, recent studies have shown that GSDME is of great importance on the mechanism of tumor cells death induced by chemotherapy (Lu et al., 2018; Wang et al., 2018; Zhang et al., 2019). HSCs are the only cells which can self-renew for life, whereas other HPCs are short-lived and committed to the transient production of mature blood cells. Under steady condition, majority of HSCs remain in a quiescent state. This property can protect HSCs from stress-induced injury and prevent their premature aging (Mohrin et al., 2010). In the present study, we found GSDME knockout led to a decrease of LSK, LT-HSC and ST-HSC, which suggested GSDME might participate in the regulation of HSC. As expected, Gsdme^−/−^ mice displayed worse survival compared with WT mice following a lethal dose of TBI. These results indicated that deletion of GSDME specifically impaired the HSCs pool in steady condition, resulting in increased sensitivity to IR. In addition, we discovered IR could inhibit the cell viability of BMCs and proliferation of HPC *in vitro*. However, there were no differences between Gsdme^−/−^ and WT mice, suggesting that GSDME knockout have no effect on the radiosensitivity of hematopoietic cells *in vitro*. The contradiction may attribute to HSC biology co-regulating by cell-intrinsic or -extrinsic regulators in the BM niche (Pinho and Frenette, 2019). GSDME serves as a switch molecule in the transformation of apoptosis and pyroptosis, and its expression level determines the mode of tumor cell death (Jiang et al., 2020). Radiation induced GSDME-mediated pyroptosis happens both in colorectal and nasopharyngeal carcinoma cells (Di et al., 2022; Tan et al., 2022). In order to elucidate whether GSDME-mediated pyroptosis is related to acute radiation injury of hematopoietic system, Gsdme^−/−^ mice and WT mice were treated with 2 Gy TBI. Increased percentages of HSC, LT-HSC, ST-HSC, HPC, NE and M cells in the spleens, accompanied by an enlarged spleen, were observed in Gsdme^−/−^ mice post IR, implying that GSDME knockout promoted extramedullary hematopoiesis. In addition, GSDME knockout could protect LSK, LT-HSC, GMP and M cells in BM, and alleviate the proliferation inhibition of HPC induced by IR. These results suggested GSDME knockout has a certain protective effect on radiation-induced acute injury of hematopoietic system which might be due to the decrease of GSDME-mediated pyroptosis, which need to be further explored.

It is well known that long-term cancer survivors are at increased risk for developing late effects related to clinical treatment, including IR-induced long-term BM injury. IR-induced long-term BM injury can deteriorate to become myelodysplastic syndrome or hypoplastic anemia over time or following additional treatment such as irradiation therapy or chemotherapy (Testa et al., 1985; Mauch et al., 1995). The long-term BM injury is mainly attributed to HSCs senescence, which is supported by the finding that HSCs isolated from sublethally irradiated mice express increased levels of senescence-associated β-galactosidase and p16. The permanent damage to HSCs ultimately results impaired self-renewal capacity, decreased long-term repopulating capacity, and myeloid skewing (Gardner et al., 2001; Shao et al., 2014). To better understand the role of GSDME in this late effect, we further tried to elucidate the role of GSDME in long-term radiation injury of hematopoietic system. Increased percentages of HPC in the spleen, accompanied by an enlarged spleen, were observed in Gsdme^−/−^ mice at 4 weeks post 4 Gy IR. However, the relative changes of hematopoietic parameters in spleens of Gsdme^−/−^ mice at 1 week post 2 Gy IR were more remarkable than those in spleens of Gsdme^−/−^ mice at 4 weeks post 4 Gy IR. Thus, these data indicated that GSDME knockout had only a slight effect on mature hematopoiesis during long-term BM injury. Additionally, GSDME knockout had a protective effect on LSK, LT-HSC, GMP, M cells, and also aggravated the damage to LY after irradiation during the long-term BM injury. These results showed GSDME deficiency had a protective effect on radiation-induced long-term injury of hematopoietic system which might be due to the radiosensitivity of GSDME knockout hematopoietic cells, ultimately leading to preferential skew toward the myeloid lineage. It has been showed that the loss of quiescence may impair the long-term repopulation capability of HSCs and eventually lead to the perturbation of hematopoietic homeostasis and self-renewal capacity (Hou et al., 2015). To further dissect the differentiation and self-renewal capacity of HSCs, we performed CRA using donors from Gsdme^−/−^ and WT mice. These results showed GSDME knockout had no protective effect on the HSCs long-term and multilineage engraftment reduction induced by IR. Taken together, our results indicate that GSDME has a regulatory effect on the maintenance of homeostasis and stress response of hematopoietic cells, but has no function on self-renewal maintenance and differentiation of HSCs.

In this study, we observed that GSDME knockout impaired HSCs pool in BM in steady condition. GSDME knockout promoted extramedullary hematopoiesis in spleens, mitigated the proliferation inhibition of HPCs, and protected HSCs and partial phenotypic myeloid hematopoietic cells (progenitor cells and M cells) after irradiation. Knockout of GSDME could also aggravate the damage to LY after irradiation during the long-term BM injury. These data indicated that GSDME knockout could alleviate both acute and long-term radiation-induced injury of hematopoietic system, and may also be related to myeloid-biased differentiation during long-term BM injury. The stress levels and degrees of tissue damage varies depending on the radiation doses of TBI. These results suggest that GSMDE plays different roles in different contexts. In steady-state, fewer HSCs in GSDME^−/−^ mice might make them more radiosensitive. But under irradiation, protecting HSCs and progenitors could aid recovery in GSDME^−/−^ mice. For the first time, we explored the role of GSDME in radiation injury of hematopoietic system, especially at the level of hematopoietic stem cells and progenitor cells. Previous study shows that GSDME is cleaved by caspase-3 to generate its pore-forming N-terminal fragment, which drives pyroptosis by disrupting plasma membrane integrity (Wang et al., 2017). In irradiated HSCs, we proposed that this pathway contributes to cell death and functional decline. While we initially aimed to validate pyroptosis markers (e.g., GSDME cleavage, IL-1β release, and membrane pore formation via microscopy/Western blot), technical constraints hindered these experiments. Specifically, primary hematopoietic cells-particularly HSCs-are notoriously difficult to culture *in vitro* due to their stemness and rapid differentiation *ex vivo*, limiting the detection of hematopoietic cell pyroptosis. Elucidating the interplay between GSDME, pyroptosis, and HSC differentiation is critical. While our current data focus on phenotypic outcomes, we are committed to defining the molecular drivers in our follow-up studies. These efforts will clarify how GSDME loss protects HSCs from irradiation injury while inadvertently skewing lineage commitment through inflammatory or epigenetic mechanisms.

In conclusion, GSDME may play a vital role in the survival and differentiation of HSCs, and its knockout aggravates myeloid-biased differentiation after irradiation. Our findings causally implicate the protection of GSDME knockout on radiation injury, thereby revealing GSDME might be a critical determining factor of radiosensitivity and serve as a potential target for radiation protection. Pharmacological blockade of caspase-3 (e.g., Z-DEVD-FMK) and IL-1β might replicate the protective effects of GSDME knockout, thereby reducing the side effects of radiation therapy.

## Data Availability

The raw data supporting the conclusions of this article will be made available by the authors, without undue reservation.
