# Supplementary material for: GSDME knockout alleviates hematopoietic stem cell irradiation injury and aggravates myeloid-biased differentiation
Source: Front Cell Dev Biol. 2025 Apr 4;13:1544320. doi: 10.3389/fcell.2025.1544320 (PMC12006135; doi:10.3389/fcell.2025.1544320)
Supplement: Supplementary file 1 [file DataSheet1.doc]

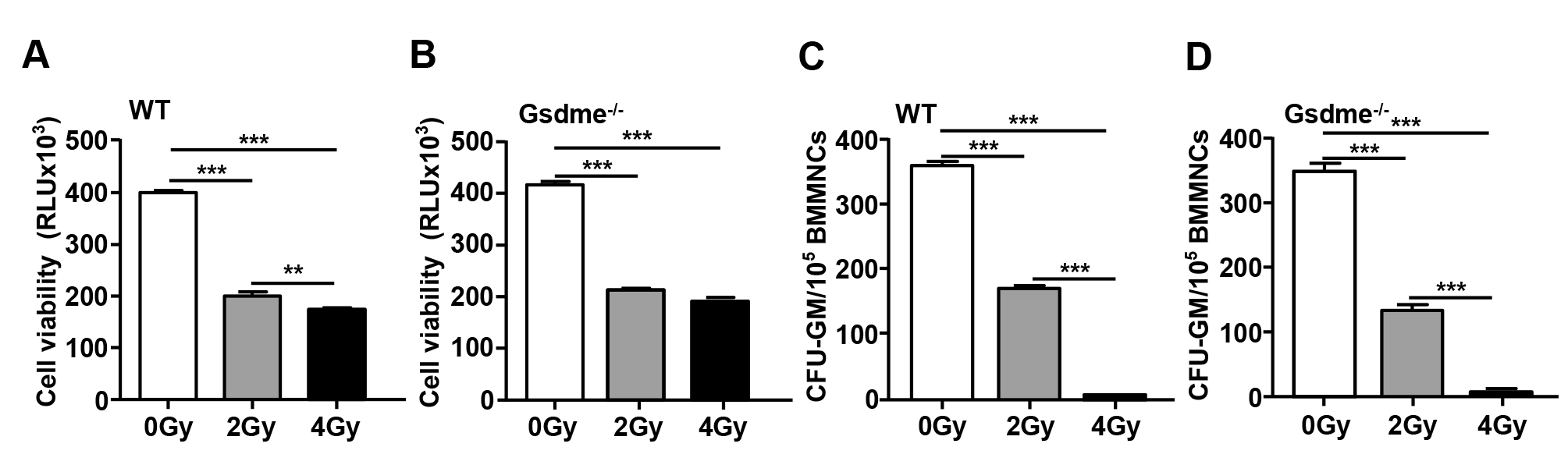


## Supplementary Figure 1. GSDME knockout had no effect on the decrease in BM cell viability or proliferation of HPCs induced by IR in vitro. (A-B) BM-MNCs harvested from WT and Gsdme^-/-^ mice at 8-12 weeks of age were plated into 96-well plates and irradiated with the needed doses (0 Gy, 2 Gy and 4 Gy) of X radiation for the cell survival assay at a dose rate of 1.08 Gy/min. After irradiation, the BM-MNCs were cultured in 5% CO2 at 37°C for 18 h, after which the cell viability was analyzed. The data were obtained from 1 experiment with 3 mice and 9 biological replicates. (C-D) BM-MNCs harvested from WT and Gsdme-/- mice at 8-12 weeks of age were irradiated with the needed doses (0 Gy, 2 Gy and 4 Gy) in vitro and cultured in MethoCult GF M3534 methylcellulose medium for 7 days, after which the CFUs of GM were analyzed. The data were obtained from 1 experiment with 3 mice and 6 biological replicates. The data are expressed as the mean ± SEM, ^**^*P*<0.01, ^***^*P*<0.001.


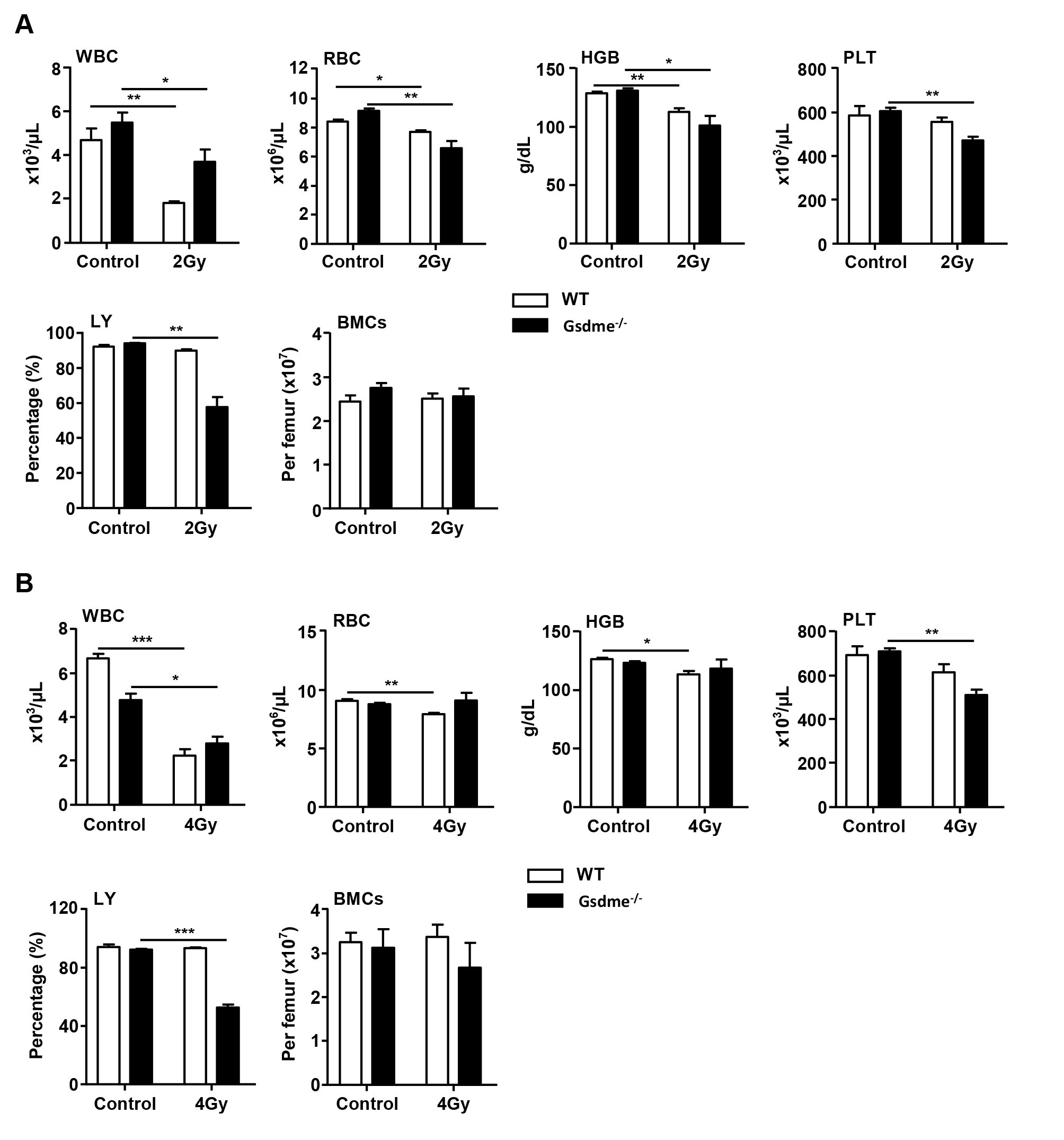


**Supplementary Figure 2. GSDME knockout had no effect on the decrease in the number of most peripheral blood (PB) cells during acute or long-term BM injury.** WT and Gsdme^-/-^ mice were exposed to IR at doses of 0 Gy, 2 Gy or 4 Gy. The PB was collected at 1 and 4 weeks after IR and in controls. PBs from the orbital sinus were collected in tubes with the anticoagulant K3EDTA. The following measurements were recorded: white blood cell (WBC) counts, red blood cell (RBC) counts, hemoglobin (HGB) levels, platelet (PLT) counts, and percentages of neutrophils (NE), LYs and monocytes (Mo). BMCs were flushed from mouse femurs with sterile phosphate buffer solution. The number of viable BMCs was calculated as ×10^7^/femur. (A) The WBC, RBC, HGB, and PLT counts and the percentages of LY in the PB of the WT and Gsdme^-/-^ mice were quantified at 1 week after IR. The number of cells in the BM of femurs from WT and Gsdme^-/-^ mice was quantified at 1 week after IR; n=3. (B) The WBC, RBC, HGB, and PLT counts and the percentages of LY in the PB of the WT and Gsdme^-/-^ mice were quantified at 4 weeks after IR. The number of cells in the BM of femurs from WT and Gsdme-/- mice was quantified at 4 weeks after IR; n=3. The data are expressed as the mean ± SEM, ^*^*P*<0.05; ^**^*P*<0.01; ^***^*P*<0.001.

## Supplementary Table 1. List of antibodies used to stain various types of hematopoietic cells

| Markers | Conjugate | Catalog number | Vendor |
| --- | --- | --- | --- |
| CD11b | APC | 17-0112-82 | eBioscience |
| CD117 (c-Kit) | APC | 17-1171-83 | eBioscience |
| CD150 | APC | 17-1501-81 | eBioscience |
| Ly-6G/Ly-6C | APC | 17-5931-82 | eBioscience |
| CD3e | APC | 17-0031-82 | eBioscience |
| CD3e | FITC | 11-0031-82 | eBioscience |
| CD34 | FITC | 11-0341-85 | eBioscience |
| CD45.1 | FITC | 11-0453-82 | eBioscience |
| CD117 (c-Kit) | PE | 12-1171-82 | eBioscience |
| CD45.2 | PE | 12-0454-82 | eBioscience |
| Ly-6A/E (Sca1) | PE | 12-5981-83 | eBioscience |
| CD3e | PE-Cyanine7 | 25-0031-82 | eBioscience |
| CD11b | PE-Cyanine7 | 25-0112-82 | eBioscience |
| Ly-6A/E (Sca1) | PE-Cyanine7 | 25-5981-82 | eBioscience |
| CD4 | Biotin | 13-0041-86 | eBioscience |
| CD8a | Biotin | 13-0081-86 | eBioscience |
| CD11b | Biotin | 13-0112-86 | eBioscience |
| CD45R/B220 | Biotin | 13-0452-86 | eBioscience |
| TER-119 | Biotin | 13-5921-85 | eBioscience |
| Ly-6G/Ly-6C | Biotin | 13-5931-86 | eBioscience |
| Streptavidin | PerCP-Cy™5.5 | 551419 | BD Biosciences |
| CD45R/B220 | PerCP | 553093 | BD Biosciences |
| CD48 | APC-Cy™7 | 561242 | BD Biosciences |
| CD127 | PE-Cyanine7 | 560733 | BD Biosciences |
| CD16/CD32 | BV421 | 562896 | BD Biosciences |
